# A216 MODERATE TO SEVERE ACUTE UPPER GASTROINTESTINAL BLEEDING IN RURAL NORTHERN ONTARIO: AN EPIDEMIOLOGICAL ANALYSIS OF AETIOLOGIES, GEOGRAPHIC LIMITATIONS, AND OUTCOMES

**DOI:** 10.1093/jcag/gwad061.216

**Published:** 2024-02-14

**Authors:** M Saunders, D Savage, P Zezos

**Affiliations:** Internal Medicine, NOSM University, Sudbury, ON, Canada; Emergency Department, Thunder Bay Regional Health Sciences Centre,, Thunder Bay, ON, Canada; Division of Gastroenterology, Thunder Bay Regional Health Sciences Centre,, Thunder Bay, ON, Canada

## Abstract

**Background:**

Hospitalizations for upper gastrointestinal bleeds (UGIBs) are resource-demanding as they often require blood transfusion, endoscopy, and intensive care unit admission. Referral centers that provide higher levels of care to remote regions treat illnesses, including UGIB, in much later stages of disease and of higher severity. The Thunder Bay Regional Health Sciences Center (TBRHSC) is a referral center servicing over 250,000 people over a geographically expansive region of Northern Ontario. Despite known barriers to accessing care, little data is currently available to guide our understanding of UGIB aetiologies, severity, and outcomes among patients living remotely in Canada.

**Aims:**

This retrospective study aimed to evaluate epidemiological data among patients with UGIB at TBRHSC.

**Methods:**

Of 441 patients with a discharge diagnosis of gastrointestinal hemorrhage from 2016–2022, inclusion criteria were met for 333. Patients under 18 y or with lower GIB were excluded. Patients were classified as regional if their primary address was outside the District of Thunder Bay.

**Results:**

Patients were highly comorbid (median CCI = 5) and presented with a median (IQR) GBS of 9 (6) and Clinical Rockall Score of 3 (1). Of those who received inpatient endoscopy (n=248), findings included erosive gastritis (21%), gastric ulcer (14%), esophagitis (12%), duodenal ulcer (8%), AVM (7%), and variceal (6%). No definite lesion was observed in 62 patients (22%). The median (IQR) Complete Rockall Score was 4 (2) with major stigmata of recent hemorrhage found in 20%. There was a high prevalence (19%) of cirrhosis, of which 83% had a history of liver decompensation. In 49% of cirrhotic patients, culprit lesions did not include portal hypertensive gastropathy or variceal bleeding. Regional patients accounted for 14% of the population and travelled an average of 424 km to seek care at TBRHSC. Regional patients were younger and required more ICU admissions (*p*=0.01). ICU admission primarily due to UGIB was 5.4% and all-cause thirty-day mortality was 3.3%. Oral anti-coagulation use prevalence was 43.2% and was associated with a longer length of stay (*p*ampersand:003C0.0001), higher proportion of all-cause 30-day mortality (*p*ampersand:003C0.005). Forty-eight patients did not receive endoscopy due to bleeding stability, palliation, and preference.

**Conclusions:**

Patients with UGIB at TBRHSC are a highly multimorbid population with a significantly burden of anticoagulant use, cirrhosis, and variceal bleeding. Comparative analysis with metropolitan centers will expand our understanding for providing UGIB care in Northern Ontario.

**Funding Agencies:**

NOAMA

